# Risk assessment in the first fifteen minutes: a prospective cohort study of a simple physiological scoring system in the emergency department

**DOI:** 10.1186/cc9972

**Published:** 2011-01-18

**Authors:** Tobias M Merz, Reto Etter, Ludger Mende, Daniel Barthelmes, Jan Wiegand, Luca Martinolli, Jukka Takala

**Affiliations:** 1 Department of Intensive Care Medicine, Bern University Hospital and University of Bern, Freiburgstrasse, 3010 Bern, Switzerland; 2 Department of Emergency Medicine, Bern University Hospital and University of Bern, Freiburgstrasse, 3010 Bern, Switzerland

## Abstract

**Introduction:**

The survival of patients admitted to an emergency department is determined by the severity of acute illness and the quality of care provided. The high number and the wide spectrum of severity of illness of admitted patients make an immediate assessment of all patients unrealistic. The aim of this study is to evaluate a scoring system based on readily available physiological parameters immediately after admission to an emergency department (ED) for the purpose of identification of at-risk patients.

**Methods:**

This prospective observational cohort study includes 4,388 consecutive adult patients admitted via the ED of a 960-bed tertiary referral hospital over a period of six months. Occurrence of each of seven potential vital sign abnormalities (threat to airway, abnormal respiratory rate, oxygen saturation, systolic blood pressure, heart rate, low Glasgow Coma Scale and seizures) was collected and added up to generate the vital sign score (VSS). VSS_initial _was defined as the VSS in the first 15 minutes after admission, VSS_max _as the maximum VSS throughout the stay in ED. Occurrence of single vital sign abnormalities in the first 15 minutes and VSS_initial _and VSS_max _were evaluated as potential predictors of hospital mortality.

**Results:**

Logistic regression analysis identified all evaluated single vital sign abnormalities except seizures and abnormal respiratory rate to be independent predictors of hospital mortality. Increasing VSS_initial _and VSS_max _were significantly correlated to hospital mortality (odds ratio (OR) 2.80, 95% confidence interval (CI) 2.50 to 3.14, *P *< 0.0001 for VSS_initial_; OR 2.36, 95% CI 2.15 to 2.60, *P *< 0.0001 for VSS_max_). The predictive power of VSS was highest if collected in the first 15 minutes after ED admission (log rank Chi-square 468.1, *P *< 0.0001 for VSS_initial_;,log rank Chi square 361.5, *P *< 0.0001 for VSS_max_).

**Conclusions:**

Vital sign abnormalities and VSS collected in the first minutes after ED admission can identify patients at risk of an unfavourable outcome.

## Introduction

The survival of patients admitted to an emergency department is determined by the severity of acute illness at admission [1] and the level and quality of care provided [2,3]. The high number of admissions and the wide spectrum of severity of illness characteristic of large emergency departments make immediate assessment of all patients by an emergency physician unrealistic [4,5]. Various scoring systems have been proposed for identification of patients at risk of deterioration of vital organ functions in the emergency department [6-9]. Ideally, the first health care provider encountering the patient should be able to recognize the need for urgent attention within minutes of emergency department admission, without laboratory and radiological examinations or the presence of a specialized physician. Systematic checks for airway, breathing, circulation and level of consciousness are included in resuscitation and trauma guidelines [10,11], and for assessment of risk of deterioration of ward patients in medical emergency team (MET) systems [12-23]. We found in a recent retrospective study that the MET calling criteria were highly predictive of hospital outcome in patients admitted to intensive care from the emergency department [24]. Most emergency departments, including ours, do not systematically screen all patients [25]. Even if a scoring system is used, the general concern about the patient's condition, as perceived by the admitting nursing staff, serves as a trigger to expedite evaluation by an emergency physician [26,27].

The time interval until appropriate care is delivered influences outcome in myocardial infarction, stroke, and sepsis [28-32]. It is conceivable that this is also the case for other groups of critically ill patients. One reason for delayed and otherwise suboptimal care is the inability to recognize signs of organ dysfunction early enough to initiate the necessary therapeutic interventions [13,33,34].

The aim of this prospective observational study was to assess the incidence of measurable vital sign abnormalities at admission to the emergency department and the potential impact of these factors on treatment delay and outcome in a large group of unselected patients needing hospital admission. We hypothesised that a scoring system based on the established MET criteria might aid in early recognition of patients at risk of an unfavourable outcome.

## Materials and methods

### Setting

The study was performed in the Department of Intensive Care Medicine and the Department of Emergency Medicine of the Bern University Hospital, a 960-bed tertiary care referral academic medical centre, in Bern, Switzerland. The emergency department provides initial evaluation and treatment of all adult patients (age >15 years).

### Patients and study design

This prospective cohort study includes all patients admitted to our hospital via the emergency department between 11 June 2007, and 11 January 2008. Data were collected prospectively on study data collection forms during the stay in the emergency department and entered in a database created for the purpose of the study. Patients treated on an outpatient basis were not included. In cases where the data were not duplicated to the study record form by the clinical staff, the research staff extracted the data; the data collection sequence and procedure by the clinical staff remained the same. Collected data included patient demographics, time of emergency department admission and discharge, time of first assessment by a physician, and the primary cause of emergency department admission (respiratory, cardiovascular, neurological, trauma, gastrointestinal or other). The time span between admission to the emergency department and discharge was broken down into a series of time periods (0 to 15 minutes, 15 minutes to 1 hour (h), 1 to 2 h, 2 to 4 h, followed by two-hour periods up to 24 h after emergency department admission) during which the presence of vital sign abnormality was investigated. Based on published MET calling criteria [12,23] assessed parameters were respiratory rate, oxygen saturation, systolic blood pressure, heart rate, Glasgow Coma Scale (GCS), presence of a threatened airway and occurrence of seizures (Table 1). The available ED monitoring system provides values for oxygen saturation (pulse oxymetry), systolic blood pressure (sphygmomanometer), heart rate (electrocardiogram), and respiratory rate (constant current impedance pneumography). Presence of a threatened airway was defined as a necessity for intratracheal suctioning, insertion of oro- or nasopharyngeal tubes, intubation, bronchoscopy and occurrence of seizures as repeated or prolonged (>five minutes) seizures. Occurrence of each of the seven potential vital sign abnormalities (VSS criteria) was considered as one VSS point, and the VSS score was defined as the total sum of all VSS points in one time period. The original MET calling criteria contain the criterion "concern", which was not included in the VSS. "Concern" represents a subjective rating rather than a measurable parameter and was shown to have a low frequency and lack of predictive value in one retrospective study in emergency patients [24]. To evaluate associations between VSS scores and predefined outcome variables, the following definitions were used: VSS_initial _denotes the VSS score in the first 15 minutes after admission to the emergency department and VSS_max _denotes the maximum VSS score throughout the total stay in the emergency department. Hence, VSS_max _represents the highest sum of VSS criteria occurring simultaneously.

**Table 1:** Vital Sign Scoring parameters

**Airway**	
• *threatened airway*:	necessity for intratracheal suctioning, insertion of oro- or nasopharyngeal tubes, intubation, bronchoscopy
**Breathing**	
• *respiratory rate:*	respiratory rate <6/minute or >36/minute
• *oxygen saturation*:	SaO_2 _<90% despite supplementary oxygen
**Circulation**	
• *systolic blood pressure:*	systolic blood pressure <90 mmHg
• *heart rate:*	heart rate <40/minute or >140/minute
**Neurology**	
• *GCS:*	Glasgow Coma Scale (GCS) score <13
• *seizures:*	repeated or prolonged (>5 minutes) seizures

Vital Sign Scoring parameters were based on medical emergency team calling criteria, as defined by Buist *et al. *and Cretikos *et al. *[12,23].

### Evaluated predictors and outcome measures

Occurrence of vital sign abnormality at emergency department admission and during emergency department stay as measured by VSS, time delay between emergency department admission, and first assessment by an emergency physician, as well as the length of stay in the emergency department, were evaluated predictors. The primary outcome measure was hospital mortality; this information was extracted from the hospital database. Secondary outcome was the combined endpoint ICU admission or death in ED. The combined endpoint was chosen to account for the fact that death occurring in the ED before discharge to the ICU was proportionately more frequent in patients with high VSS than in patients with low VSS.

Missing data: In cases where data on vital signs were not entered in the study data collection forms, these data were extracted from the ED patient charts or anaesthesia charts. To analyze potential bias between patients with missing data and the rest of the cohort, age, hospital mortality and VSS scores of these patients were compared with patients whose complete data were collected on the study forms.

### Ethical approval and patient consent

The study was approved by the Ethical Committee of the Canton of Bern, and adheres to the tenets of the Declaration of Helsinki. The need for informed consent was waived provided that purely observational data were collected in conjunction with the normal clinical management. Nevertheless, all patients admitted to the Bern University Hospital are routinely informed of their right to specify whether data related to their stay can be used in observational studies; data of patients who declined were not included in the study.

### Statistical analysis

The data were not normally distributed, and are presented as median and interquartile ranges. Comparison of outcome groups defined on the basis of hospital survival/non-survival was performed using the non-parametric Mann-Whitney test or the Chi-square test, as appropriate. Survival in different groups, defined by the primary cause of emergency department admission, was analyzed by applying categorical logistic regression. The predictive value of VSS_initial _and VSS_max_, in relation to hospital mortality was assessed by univariate logistic regression. To assess survival differences throughout the whole score range groups stratified by VSS scores were compared pair-wise using Pearson's Chi square test. Additionally, Kaplan-Meier survival plots were constructed and log rank and Chi-square tests were used to compare survival in groups stratified by VSS_initial _and VSS_max_. Subjects were censored at the time of hospital discharge. Additionally, receiver operating characteristic (ROC) curves were constructed and the area under the curve (AUC) was calculated to assess the capability of VSS_inital _to discriminate survivors from non-survivors. The prognostic significance of an increase of the VSS score during the stay in the emergency department was assessed in a multivariate logistic regression model including VSS_initial _and the increase in VSS points (VSS_max _- VSS_initial_) as predictors and hospital mortality as outcome parameter. Pearson's Chi-square test was used to assess the value of single VSS criteria with regard to hospital mortality. The results of the single Chi-square tests were compared using Cramer's V (values ranging from 0 to 1, with 0 = no association between variables and 1 = complete association of variables). Forced entry multivariate logistic regression analysis, with all covariates into the regression model in one block, was used to identify independent predictors of mortality. The correlations between VSS_initial _scores, the delay until the first assessment of an emergency physician, and length of stay (LOS) in the emergency department and hospital mortality were assessed in univariate and multivariate logistic regression models, as indicated. The correlation between VSS_initial _and the delay until the first assessment of an emergency physician was assessed using linear regression. In all analyses a *P*-value of 0.05 or less was considered statistically significant. Statistical analyses were performed using the software packages SPSS version 13.0 (SPSS, Inc., Chicago, IL, USA) and GraphPad Prism version 4.02 (GraphPad Software, San Diego, CA, USA).

## Results

### Patient characteristics

**Figure 1:**
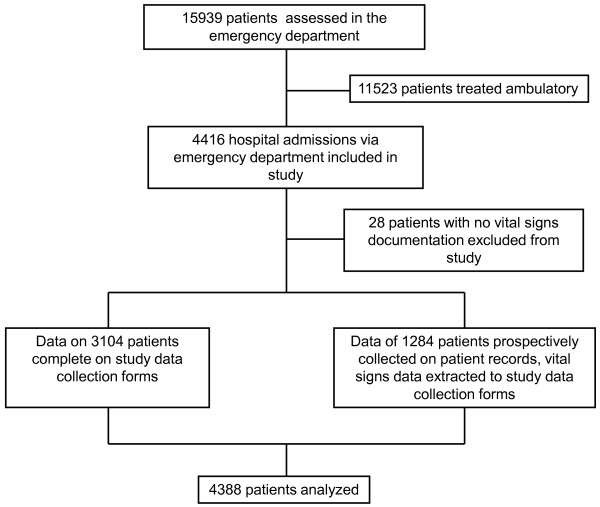
**Study flow chart**. Flow chart of patients included in study.

A total of 4,416 emergency hospital admissions through the emergency department occurred during the study period. Data on 3,104 patients were collected and entered into their study forms during their stay in the ED. In 1,284 patients, data had to be extracted from the ED patient charts. In 28 patients (0.6%), study data on vital sign abnormality were not available; these patients were excluded from the analysis. Thus, a total of 4,388 patients with an overall hospital mortality of 7.2% were studied (Figure 1). Non-survivors were significantly older and had higher VSS_initial _and VSS_max _scores than surviving patients. The primary cause of emergency department admission was not correlated with hospital mortality. Non-surviving patients had significantly shorter emergency department and hospital length of stay and were assessed with less time delay by an emergency physician (Table 2). Table 3 summarizes the number of patients and hospital mortality per VSS_initial _and VSS_max _scores.

**Table 2:** Patient characteristics in groups stratified by hospital outcome

	All patients	Hospital survivors	Hospital non-survivors	*P*-value
Number of patients	4,388	4,072	316	
Age	61.0 (44.3 to 74.1)	60.3 (43.0 to 73.5)	69.6 (57.3 to 79.7)	<0.0001
VSS_max _(points; median/IQR)	0 (0 to 1)	0 (0 to 0)	1 (0 to 2)	<0.0001
VSS_initial _(points; median/IQR)	0 (0 to 0)	0 (0 to 0)	1 (0 to 2)	<0.0001
				
Primary cause of emergency department admission (% of patients)				0.078
Respiratory	333(7.0)	295 (7.2)	38 (5.7)	
Cardiovascular	633 (13.4)	558 (13.7)	75 23.7)	
Neurological	895 (18.9)	832 (20.4)	63 (19.9)	
Trauma	815 (17.2)	776 (19.1)	39 (12.3)	
Gastrointestinal	607(12.8)	570 (14.0)	37 (11.7)	
Other	1,105 (23.3)	1,041 (25.6)	64 (20.3)	
delay first physician (hours; median/IQR)	0.17 (0.0 to 0.5)	0.17 (0 to 0.51)	0.08 (0 to 0.41)	<0.0001
length of emergency department stay (hours; median/IQR)	4.6 (2.8 to 7.3)	4.6 (2.9 to 7.4)	4.1 (1.6 to 6.6)	<0.0001
length of hospital stay (days; median/IQR)	6.3 (3.0 to 11.8)	6.5 (3.1 to 11.8)	3.4 (0.7 to 11.4)	<0.0001

IQR, interquartile range; VSS, Vital Sign Score.

**Table 3:** Number of patients and hospital mortality in groups stratified by VSS_initial _and VSS_max _scores

	**VSS**_**initial**_		**VSS**_**max**_	
	Number of patients (%)	Hospital mortality	Number of patients (%)	Hospital mortality
VSS 0	3,625 (82.6%)	3.9%	3,217 (73.3%)	3.6%
VSS 1	490 (11.2%)	13.9%	577 (13.1%)	11.6%
VSS 2	167 (3.8%)	25.1%	450 (10.3%)	13.1%
VSS 3	58 (1.3%)	43.1%	79 (1.8%)	36.7%
VSS ≥ 4	48 (1.1%)	79.2%	65 (1.5%)	69.2%

VSS, Vital Sign Score

### Survival analysis of VSS scoring

**Figure 2:**
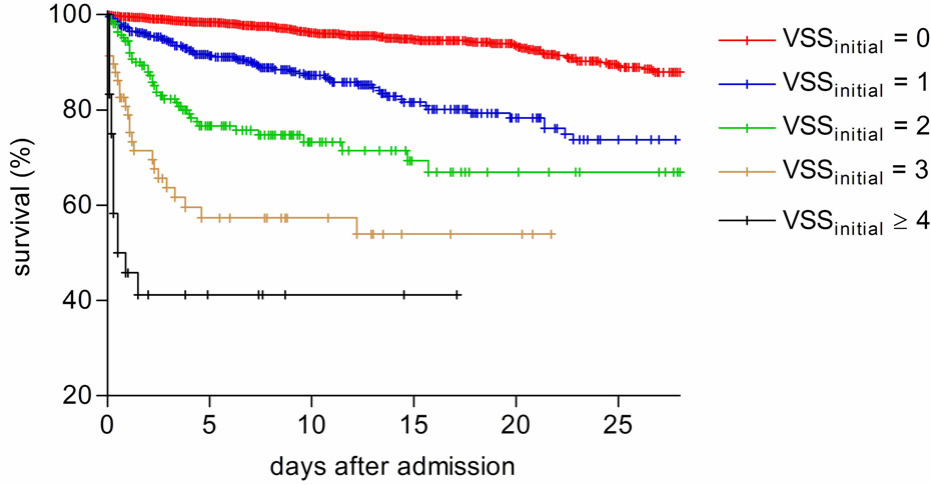
**Hospital survival in the strata of VSS_initial _groups**. Kaplan-Meier plot of hospital survival in the strata of VSS_initial _groups (log rank Chi-square 468.1, *P *< 0.0001).

**Figure 3:**
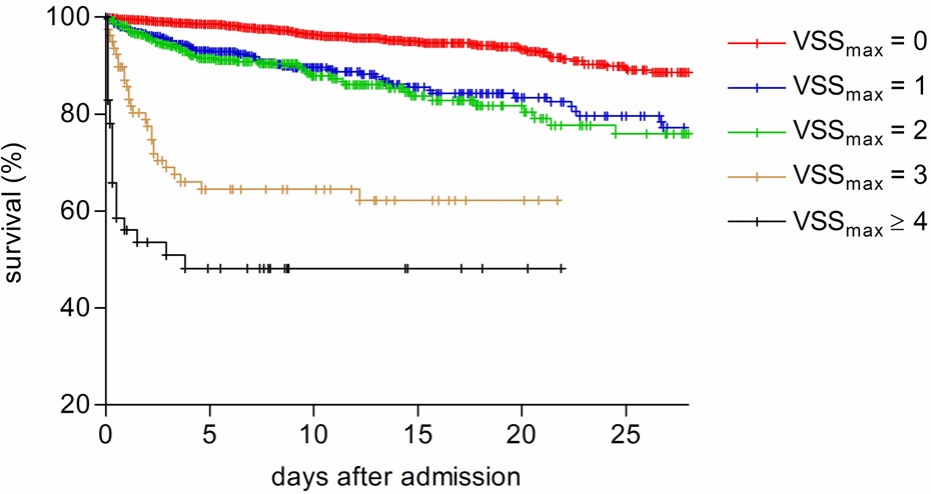
**Hospital survival in the strata of VSS_max _groups**. Kaplan-Meier plot of hospital survival in the strata of VSS_max _groups (log rank Chi square 361.5, *P *< 0.0001).

**Figure 4:**
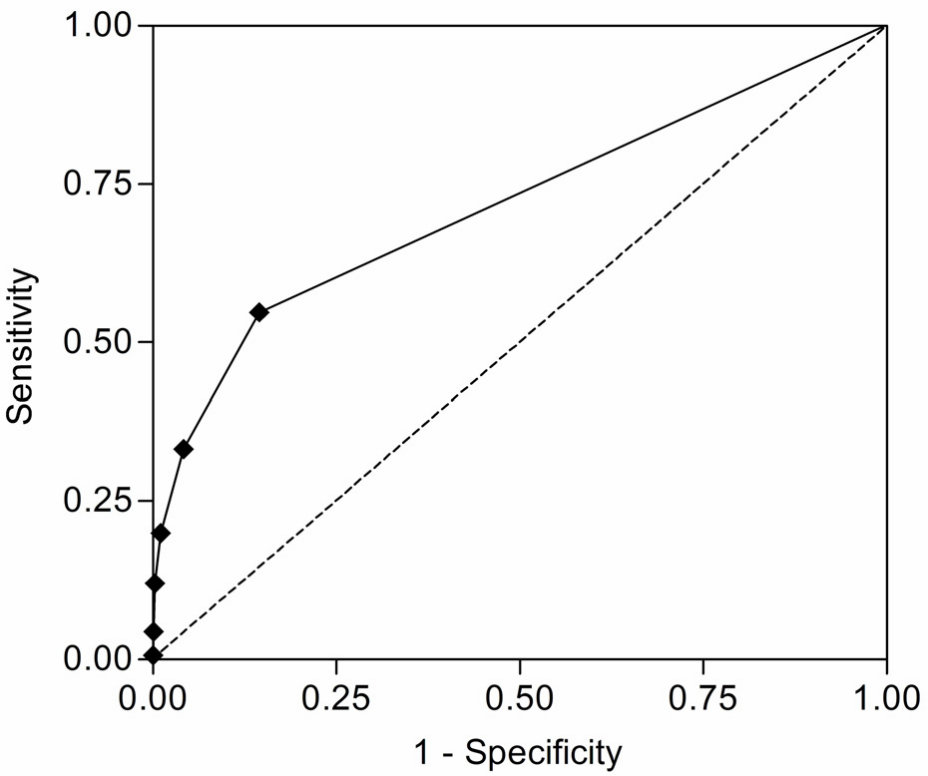
**ROC curve for VSS_initial_**. Receiver operating characteristic curve for VSS_initial _in relation to hospital survival. The area under the curve was 0.72 (95% CI 0.53 to 0.91, *P *< 0.0001).

VSS_initial _and VSS_max _were both predictors of hospital survival odds ratio (OR) 2.80, 95% confidence interval (CI) 2.50 to 3.14, *P *< 0.0001 for VSS_initial_; OR 2.36, 95% CI 2.15 to 2.60, *P *< 0.0001 for VSS_max_). The prognostic accuracy of VSS_initial _in predicting hospital outcome was superior to VSS_max _(log rank Chi-square 468.1, *P *< 0.0001 for VSS_initial_; log rank Chi square 361.5, *P *< 0.0001 for VSS_max_) (Figures 2 and 3). For VSS_initial_, survival differences were significant over the whole score range except for VSS_initial _3 and 4; for VSS_max _the difference between scores 1 and 2 was not significant (Table 4). Vital sign instabilities developed or increased in 516 patients while in the emergency department (VSS_max _> VSS_initial_). These patients had a higher mortality than patients in whom the VSS score was highest at admission (OR 1.49, 95% CI 1.09 to 2.05, *P *= 0.015). Figure 4 shows the ROC curve for VSS_initial _plotting sensitivity versus 1-specificity. The AUC was 0.72 (95% CI 0.53 to 0.91, *P *< 0.0001), indicating a moderately to highly predictive value of VSS_initial _in relation to hospital mortality.

**Table 4:** Survival differences in patient groups stratified by VSS_initial _and VSS_max _scores

VSSinitial					VSSmax			
	Chi-square	OR	95% CI	*P*	Chi-square	OR	95% CI	*P*
VSS 0/1	94.31	4.10	3.03 to 5.54	<0.0001	65.7	3.45	2.54 to 4.77	<0.0001
VSS 1/2	11.32	2.11	1.38 to 3.23	0.0008	0.89	1.22	0.84 to 1.76	0.35
VSS 2/3	13.04	3.21	1.73 to 5.97	0.0003	23.23	3.63	2.14 to 6.17	<0.0001
VSS 3/4	0.01	1.029	0.48 to 2.22	0.94	8.90	2.95	1.50 to 5.81	0.0029

VSS, Vital Sign Score

### Secondary endpoint ICU admission or death in ED

VSS_*initial *_was a significant predictor of the necessity of ICU admission or death in the ED (OR 3.14, 95% CI 2.80 to 3.52, *P *< 0.0001). The secondary endpoint was reached by 14.9% of patients with a VSS_initial _of 0; respective percentages for VSS_initial _1 to ≥4 were 33.7%, 67.7% 75.9% and 100%.

### Prognostic significance of single VSS scoring criteria

Univariate analysis revealed that all VSS_initial _criteria except for seizures were associated with hospital outcome (Table 5). In the multivariate analysis the VSS criteria GCS, systolic blood pressure and oxygen saturation were the most significant independent outcome predictors, followed by heart rate and threatened airway. The criteria respiratory rate and seizures were not independent predictors of hospital mortality (Table 6).

**Table 5:** Frequency and results of Chi-square test of single VSS_initial _criteria

**VSS**_**initial **_**parameter**	Frequency of single VSS criteria (% of all patients)	Odds ratio	Limits of 95% confidence interval		Cramer's V	*P*-value
			lower	upper		
threatened airway	159 (3.6%)	9.70	6.88	13.68	0.23	<0.0001
respiratory rate	80 (1.8%)	4.84	2.90	8.08	0.10	<0.0001
heart rate	154 (3.5%)	5.86	3.93	8.77	0.15	<0.0001
oxygen saturation	297 (6.8%)	4.61	3.41	6.21	0.16	<0.0001
systolic blood pressure	202 (4.6%)	10.96	8.04	14. 98	0.28	<0.0001
GCS score	262 (6%)	12.41	9.35	16.47	0.32	<0.0001
seizures	56 (1.3%)	0.0	0.0	0.0	0.01	0.99

GCS, Glasgow coma scale, VSS, Vital Sign Score. The results of Chi-square tests of single VSS_initial _criteria are given as odds ratio, Cramer's V (degree of association of single VSS criteria and hospital mortality; 0 denoting no association, 1 denoting maximum association) and significance value.

**Table 6:** Results of multivariate logistic regression of individual VSS criteria

**VSS**_**initial **_**parameter**	odds ratio	limits of 95% confidence interval		*P*-value
		lower	upper	
Threatened airway	1.66	1.02	2.68	0.041
Respiratory rate	0.74	0.36	1.54	0.42
Heart rate	2.37	1.45	3.86	0.001
Oxygen saturation	2.91	2.02	4.20	<0.0001
Systolic blood pressure	3.88	2.62	5.75	<0.0001
GCS score	6.18	4.20	9.08	<0.0001
Seizures	0.83	0.31	2.26	0.83

GCS, Glasgow coma scale; VSS, Vital Sign Score. Results of multivariate logistic regression of individual VSS criteria recorded in the first 15 minutes after emergency department admission, identifying independent predictors given as odds ratio, 95% confidence interval of odds ratio and significance value for hospital mortality.

### Correlations between scores, delay to first assessment and LOS in the emergency department and hospital mortality

The delay between emergency department admission and the first assessment by an emergency physician was not a predictor of hospital mortality in a univariate analysis (OR 0.99, 95% CI 0.94 to 1.04, *P *= 0.69) or after correction for vital sign abnormalities at admission (VSS_initial_) (OR 0.98, 95% CI 0.94 to 1.04, *P *= 0.65). Shorter LOS in the emergency department was associated with a higher hospital mortality (OR 0.95, 95% CI 0.92 to 0.98, *P *< 0.0001). After correction for vital sign abnormalities at admission (VSS_initial_), LOS in the emergency department lost its predictive value for hospital outcome (OR 0.99, 95% CI 0.96 to 1.01, *P *= 0.25).

### Missing data

Patients with complete study form data were slightly younger (median age 59.7 vs 60.8, *P *= 0.009) but had similar hospital mortality (7.0% vs. 7.3%; *P *= 0.72) as compared to patients whose data were extracted from the patient records. There were no significant differences in the distribution of VSS_inital _groups (VSS_inital _0: 85.0% vs. 82.5%; VSS_inital _1: 7.03 vs. 12.54%, VSS_inital _2: 4.57 vs. 3.31%; VSS_inital _3: 1.97 vs. 1.11%; VSS_inital _≥4: 1.40% vs 0.48%; *P *= 0.29) between the two groups.

## Discussion

The main finding of this study was that VSS scores based on simple criteria to assess vital sign instability within the first 15 minutes of admission to the emergency department were highly predictive of hospital mortality and necessity of ICU admission in a general population of emergency department patients. The VSS allows for simple and rapid evaluation of patients immediately after emergency department admission by the first health care provider looking after the patient. It may, therefore, facilitate the triage of patients in the emergency department, help caregivers recognize those patients requiring the most urgent attention, and help to avoid delays in implementation of necessary organ function support and commencement of treatment. The sum of single vital sign instabilities is sufficient to obtain the VSS, whereas other reported triage scores [7,35,36] use weighted assessments of vital function parameters and require time-consuming calculations and the use of specific scoring tables. Even if this only takes a few minutes, it might preclude the routine use of these scores in every patient. The prognostic accuracy of the VSS was best if collected early after admission. Whereas VSS_initial _represents the patient's condition before the start of treatment, VSS_max _can represent a high score at ED admission and decrease thereafter (positive reaction to resuscitation efforts) or an increase from a lower score (deterioration despite treatment). These two different trends in the patient's condition and reaction to treatment potentially influence the patient's outcome and might explain the difference in the prognostic power of VSS_initial _and VSS_max_.

Our results emphasize that the presence, onset, or worsening of vital sign instability in the course of the emergency admission worsens hospital outcome. Not just the initial VSS score but its change during the emergency department stay is relevant: at the same VSS_initial _level, patients with increasing VSS scores had higher hospital mortality than those with an unchanged or decreased score in later assessments. We have no data on whether these patients deteriorated despite timely treatment or due to treatment delay.

Despite the various physiological triage systems available to identify at-risk patients in the emergency department outcome studies applying these triage scoring systems are scarce and available only in selected subgroups of emergency patients. The concept of adding up the VSS criteria applied in this study is analogous to the use of the sum of failing organs for the calculation of organ dysfunction scores in intensive care [37-39] and we previously used a similar approach for patients admitted to intensive care from the emergency department [24].

It is conceivable that the individual components of the VSS score may have different relevance for the subsequent clinical course. In the present study, impaired levels of consciousness, hypotension, hypoxemia, and abnormal heart rate were the strongest predictors of mortality. In our previous study on patients admitted to intensive care from the emergency department, respiratory rate, decreased level of consciousness, hypoxemia, hypotension, and abnormal heart rate within the first hour in the emergency department were the strongest predictors of mortality. In ward patients, bradypnea, tachypnea, impaired consciousness, high heart rate, low blood pressure, and high respiratory rate were predictors of mortality [40]. Despite the different patient cohorts and ranking of predictors, all these studies emphasize the relevance of decreased levels of consciousness and cardiovascular and respiratory instability as early predictors of mortality risk.

The lack of independent predictive value for seizures and respiratory rate may be regarded as surprising. Seizures have been associated with increased risk of sudden death [41]. The 56 patients with seizures in this study had a mortality of 8.9% (vs. 7.8% for the whole cohort). It is conceivable that the simultaneous presence of other VSS components (for example, hypoxemia and low GCS) may have masked the independent predictive value of seizures. The same can be assumed for respiratory rate: it is likely to have occurred in conjunction with hypoxemia, followed by immediate intubation.

The outcome of critically ill patients in the emergency department can be ameliorated by rapid identification and initiation of appropriate treatment. This is true of ill patients in general [42] and in subgroups such as septic shock [29], trauma [28], acute ischemic stroke [32] and acute myocardial infarction [30]. Optimal management of patients who require advanced organ support seems to be of particular importance, and may have a marked effect on eventual outcome [43,44]. The VSS represents a simple scoring system that allows identification of at-risk patients within minutes after arrival. Whether it facilitates rapid commencement of treatment and improves the outcome of these patients is an unanswered question which should be addressed by future research.

The main strength of our study is the use of well-established criteria for the evaluation of vital sign abnormalities to generate a simple scoring system, the prognostic value of which was prospectively assessed in patients admitted to the emergency department of a tertiary referral hospital over a period of six months. The analyzed sample size was large and represents a cohort originating from a broad (adult) population covering the whole spectrum of emergencies; all outcomes until hospital discharge were available.

The main limitations of our study are related to the single-centre design and the need to retrospectively extract missing data from patient records. Focusing our study on hospital admissions and excluding patients treated on an outpatient basis could introduce a selection bias for the study population, as the decision for admission or ambulatory treatment has not yet been made at the time a patient presents at the ED. However, the main outcome parameter of the study was hospital mortality, which can only occur in patients admitted to the hospital. Inclusion of study subjects who by definition cannot reach the main endpoint of the study would confound the results. Whether the VSS score can help to select patients who can be treated as outpatients should be studied separately. Our hospital serves as a primary care centre for a large urban area as well as a tertiary care centre for specialized evaluation and treatment of a population of approximately 1.5 million. With regard to structure and organisation our institution is comparable to other university hospitals in Switzerland and in other countries. Despite the need to extract vital signs data from the patient records in a substantial number of patients, we are confident that this has not biased the main results of the study. All the data needed for the VSS were collected by the same staff as part of their routine clinical work. In cases where the data were not duplicated to the study record form by the clinical staff the research staff extracted the data, the data collection sequence and procedure by the clinical staff were the same. Only in a very small fraction of patients (28 patients) the data for VSS were not available. Furthermore, we found no clinically relevant differences between the characteristics or outcomes in those patients where the vital sign data were collected in both the study form and the patient records vs those with data collected in the patient records only. Finally, since the data were collected without actions to alter the clinical routine, we have no reason to believe that the patients would have been treated differently.

Inter-observer variation in the accuracy of data collection was not assessed. Determination of inter-observer variation of all the involved health care professionals would not have been possible due to the limited study resources. All ED staff had to attend lectures on how to collect the required parameters correctly prior to the study commencement. Parameters were strictly defined and not study specific but part of the already implemented routine clinical data collection. Most data originated from automatic monitoring systems. Therefore, we do not expect a significant bias by high inter-observer variation.

We consider the observed frequency of vital sign instability as a minimum prevalence, since the vital signs were recorded as part of the clinical routine. It is conceivable that the use of continuous monitoring technologies and protocols triggering changes in routine monitoring and treatment based on the observed abnormalities may alter both the detection and occurrence rate of vital sign abnormalities. Finally, only if the detection of vital sign abnormalities triggers the correct intervention can an improvement of outcome be expected. We suggest that the VSS provides a pragmatic approach for structured detection of outcome-relevant vital sign abnormalities and a tool for interventional studies.

## Conclusions

In this prospective cohort study we found that in patients admitted to the emergency department, a score derived from readily available physiological parameters registered during the first 15 minutes after admission was strongly associated with the subsequent risk of death. The use of the VSS score in the emergency department may help to design interventions for faster and more systematic identification and treatment of patients at risk of an unfavourable outcome and to avoid delays in implementing organ function support.

## Key messages

• A score (Vital Sign Scoring; VSS) derived from simple criteria to assess vital sign instability within the first 15 minutes of admission to the emergency department is highly predictive of hospital mortality.

• The VSS allows for simple and rapid evaluation of patients immediately after emergency department admission by the first health care provider looking after the patient.

• The use of the VSS in the emergency department may help to design interventions for faster and more systematic identification of patients at risk of an unfavorable outcome.

• The VSS may help to avoid delays in treatment and implementation of organ function support in critically ill patients in the emergency department.

## Abbreviations

CI: confidence interval; ED: emergency department; GCS: Glasgow Coma Scale; LOS: length of stay; MET: medical emergency team; OR: odds ratio; VSS: Vital Sign Scoring.

## Competing interests

The Department of Intensive Care Medicine has, or has had in the past, research contracts with Abbott Nutrition International, B. Braun Medical AG, CSEM SA, Edwards Lifesciences Services GmbH, Kenta Biotech Ltd, Maquet Critical Care AB, Omnicare Clinical Research AG, and Orion Corporation; and research and development/consulting contracts with Edwards Lifesciences SA, Maquet Critical Care AB, and Nestlé. The money is/was paid into a departmental fund; no author receives/received individual fees. These contracts are unrelated to and did not influence the current study.

## Authors' contributions

TM, RE, LMe, LMa and JT participated in the design of the study. DB designed the study database. RE, DB, LMe and LMa collected all data on ED patients. TM and DB performed the statistical analysis. The manuscript was drafted by TM, assisted by JW and JT. All authors read and revised the manuscript drafts and approved the final manuscript.
